# Efficacy of Acupuncture for Primary Insomnia: A Randomized Controlled Clinical Trial

**DOI:** 10.1155/2013/163850

**Published:** 2013-09-18

**Authors:** Jing Guo, Lin-Peng Wang, Cun-Zhi Liu, Jie Zhang, Gui-Ling Wang, Jing-Hong Yi, Jin-Lian Cheng

**Affiliations:** Acupuncture and Moxibustion Department, Beijing Hospital of Traditional Chinese Medicine Affiliated to Capital Medical University, 23 Meishuguanhou Street, Beijing 100010, China

## Abstract

*Objectives*. To investigate the six-week influence of acupuncture on sleep quality and daytime functioning in primary insomnia. *Methods*. The study was a double-dummy, single-blinded, randomized, placebo-controlled clinical trial. A total of 180 patients with primary insomnia were randomly assigned to 3 groups: verum group underwent verum acupuncture plus placebo; estazolam group underwent estazolam plus sham acupuncture; sham group underwent sham acupuncture plus placebo. The outcome was measured by Pittsburgh Sleep Quality Index (PSQI), Epworth Sleepiness Scale (ESS), and the 36-item short-form health survey (SF-36). *Results*. The three groups showed significant improvement compared with the pretreatment baseline. Compared with the other two groups, the verum group reported improved sleep quality (SQ) and vitality (VT), decreased daytime dysfunction (DD) and sleepiness (ESS score). The differences were kept from the treatment period to the end of the trial. *Discussion*. Verum acupuncture appeared to be more effective in increasing sleep quality and daytime functioning than sham acupuncture and estazolam. *Trial Registration*. The trial is registeded with ClinicalTrials.gov ISRCTN12585433.

## 1. Introduction

Insomnia is a common clinical complaint. The predominant features are difficulty initiating or maintaining sleep or nonrestorative sleep. Sleep disturbance causes clinically significant distress or impairment in social, occupational, or other important areas of functioning [1].

About 23.2% of adult population in the United States experiences insomnia [2]. The prevalence of insomnia ranges from 11.7% to 37% in some European countries [3–5], 9.2% to 11.9% in Asia [6–8]. The prevalence varies considerably depending on the definition used. When daytime consequences of insomnia are taken into account, the prevalence is between 9% and 15% [9].

Disorders of the sleep-wake cycle have negative impact on daytime functioning. It is considered to be associated with fatigue, sleepiness, decreased alertness, concentration and mood disturbances, and so forth [10–15], although there still remains discrepancy about how to assess daytime impairments objectively [16–19]. In the long run, daytime deficits heighten the risk of absenteeism, impaired work performance [2, 20–22], and higher odds for automobile accidents [23, 24]. The disturbances have resulted in high economic burden [25, 26]. 

For chronic insomnia, hypnotic medications (benzodiazepine receptor agonists, in particular) and cognitive-behavioral therapy (CBT) are first-line treatments. Benzodiazepine receptor agonists (BZRAs) are efficacious in the short-term management of insomnia. But there is very limited evidence of the long-term treatment efficacy of these agents [27]. They are also related with the adverse effects of residual daytime sedation, cognitive impairment, dependence, and so forth. CBTs have demonstrated efficacy in randomized clinical trials (RCTs). However, these techniques are not widely used due to lack of trained therapists [28]. The guidance for clinicians in choosing the best treatment is limited so far. 

As an alternative therapeutic method, acupuncture offers another option for insomnia. It is based on the theory of meridians of Traditional Chinese Medicine (TCM). Meridian is considered to be a network of passages of the energy power, Qi. According to ancient TCM classic of Nei Jing (Inner Classic), insomnia is a consequence of the vicious cycle of “daytime low-spirit” and “nighttime hyperarousal state.” Acupuncture is considered to be beneficial to restore the normal sleep-wake cycle by regulating and restoring the natural flow of Qi. That may explain why acupuncture is usually conducted in daytime but not at night. 

Acupuncture is one of the most common therapies for insomnia in China. Nevertheless, its evidence is plagued by methodology design limitation. Ten systematic reviews between 2003 and 2010 on acupuncture treatment of insomnia drew different conclusions. Their results were far from uniform. Only two reviews suggested that acupuncture was beneficial for insomnia [29]. More high methodological quality clinical trials are needed to further study the efficacy of acupuncture for insomnia. 

Most of the clinical trials of acupuncture for insomnia focused on the effects of sleep quality; however, daytime functioning was not highly considered. The National Institutes of Health has emphasized the analyses including measures of sleep, daytime functioning, and quality of life [28]. We have conducted a small sample pretest on the influence of acupuncture on daytime functioning and sleep quality of insomnia [30]. The study suggested that insomnia sufferers were usually more energetic at daytime when they undertook acupuncture. 

Based on the results from our previous pilot study, we designed a randomized controlled trial to investigate the efficacy of verum acupuncture, estazolam, and sham acupuncture on sleep quality and daytime functioning for insomnia. 

## 2. Methods

### 2.1. Design

This trial was randomized, double-dummy, single-blinded, and placebo-controlled. It compared the efficacy of verum acupuncture, estazolam, and sham acupuncture for insomnia. Outcome measurements were assessed at baseline, posttreatment period, and 2-month follow-up. The trial was performed according to the principles of the Declaration of Helsinki (Version Edinburgh 2000). The protocol was approved by the Medical Ethical Committee of Beijing Hospital of Traditional Chinese Medicine affiliated to Capital Medical University (Beijing TCM hospital) in August 2009. 

### 2.2. Participants

The patients were recruited mainly by hospital-based advertisements from out-patient clinic of Beijing TCM hospital between August 2009 and May 2011. The inclusion criteria were (1) aged 25–75 years; (2) diagnosed from Diagnostic and Statistical Manual of Mental Disorders-Text Revision, 4th ed (DSM-IV-TR); (3) experienced insomnia for 4 weeks or longer before the start of observation period; (4) not yet received any psychoactive medications. 

The exclusion criteria were (1) having depression, anxiety or schizophrenia; (2) diagnosis of serious disease of heart, brain, kidney, or liver; (3) history of sleep apnoea; (4) treatment with investigational drugs in the past six months; (5) ever having acupuncture for insomnia or receiving acupuncture for any indication during the last year; (6) pregnancy, breast-feeding.

After the specified assessor's evaluation, subjects who met the inclusion criteria were instructed that they would be randomly assigned to the verum acupuncture group, the sham group, or the estazolam group. Patients intending to the trial obtained informed consent. Subjects in the sham group were given the choice of extending 4 weeks of treatment with verum acupuncture free of charge after the completion of the trial.

### 2.3. Sample Size

Based on our previous pilot study of acupuncture for primary insomnia [30], Epworth Sleepiness Scale (ESS) score decrease was 5.19 ± 3.81 in the acupuncture group and 1.90 ± 3.93 in the control group. The difference was statistically significant. Based on 0.9 power to detect a significant difference (*α* = 0.05, two-sided), 50 patients were required for each group. To compensate for a dropout rate of 20%, 60 patients per group were recruited.

### 2.4. Randomization and Allocation

The computerized randomization scheme was designed by Research Center of Clinical Epidemiology Affiliated to Peking University. The random allocation sequence was generated with a block of 6. Patients' screening sequence numbers were printed outside the envelope, and the group names were printed inside. All envelopes were numbered in sequential order. Then the subjects were randomly assigned to the three groups in a 1 : 1 : 1 ratio. 

### 2.5. Blinding

Patients were blinded to the type of acupuncture and the medicine they received. A double-dummy method was adopted to raise the degree of blindness. The efficacy of verum acupuncture plus placebo drug, estazolam plus sham acupuncture, and placebo drug plus sham acupuncture was compared in the trial. In addition, outcome assessors and statistician were blinded to the group assignments. Due to the procedure of the acupuncture technique, it was not possible to blind the acupuncturists.

### 2.6. Intervention

#### 2.6.1. Verum Acupuncture Group

Subjects assigned to the verum group were needled at the points of Shenting (DU-24), Sishencong (EX-HN1), Baihui (DU-20), Sanyinjiao (SP-6), and Shenmen (HT-7) using stainless steel needles (0.32 × 40 mm, HuaTuo, China). The acupoints selection was based on our previous study on primary insomnia [30], literature review [31], and the experts' experience in treating insomnia [32]. Baihui (Du-20), Shenting (DU-24), and Sishencong (EX-HN1) were punctured at a depth of 10 mm obliquely. Sanyinjiao (SP-6) was punctured 10 mm straightly and Shenmen (HT-7) was inserted 5 mm perpendicularly. Needle manipulation, that is, lifting and thrusting, rotating or twirling, was applied to achieve “De Qi,” a needle sensation of feelings of soreness, numbness, fullness, burning, heaviness, aching, and so forth, based on subjective reporting of the patients [33]. Needles retention was 30 minutes. The acupuncture was performed every other day for six weeks.

One estazolam placebo tablet was taken 30 min prior to bedtime in the day without acupuncture intervention. The placebo medicines were produced by Beijing Yimin Pharmaceutical Co, Ltd. It had exactly the same appearance as true estazolam.

#### 2.6.2. Estazolam Group

In the estazolam group, subjects were treated with estazolam and sham acupuncture for six weeks. Estazolam (1 mg) was given 30 min prior to bedtime every other day. In the day without estazolam intervention, sham acupuncture was conducted by needling the acupoints of Binao (LI-14), Shousanli (LI-10), Yuji (LU-10), and Fengshi (GB-31). According to lecture review and clinical experiences, the acupoints were mainly used for local disease and having no therapeutic effect for insomnia.

Stainless steel needles of the same specifications were inserted superficially at the acupoints and kept for 30 minutes. Manual stimulation and De qi were avoided. 

#### 2.6.3. Sham Group

Subjects assigned to sham group were treated with sham acupuncture and estazolam placebo tablet for six weeks. Sham acupuncture treatment was the same as in the estazolam group. In the day without acupuncture intervention one estazolam placebo tablet was given 30 min prior to bedtime. 

### 2.7. Quality Control

All acupuncturists and assessors had at least 15 years of professional experience. They were required to undergo special training prior to the trial to guarantee consistent practices. The training program included diagnoses, inclusion and exclusion criteria, location of the acupoints, acupuncture manipulation techniques, and completion of case report forms (CRFs). Periodic monitoring guaranteed accuracy and quality throughout the study.

### 2.8. Outcome Measures

#### 2.8.1. Sleep Measures

PSQI is a self-rated questionnaire which assesses sleep quality and disturbances. Nineteen individual items generate seven “component” scores: subjective sleep quality (SQ), sleep-onset latency (SOL), total sleep time (TST), habitual sleep efficiency (SE), sleep disturbances (Dyssomnia), use of sleeping medication, and daytime dysfunction (DD) [34]. Since the medicine was limited in the trial, the component score was omitted.

#### 2.8.2. Daytime Functioning

Epworth Sleepiness Scale (ESS) is a simple, self-administered questionnaire designed to measure the subject's genera1 level of daytime sleepiness [35]. It can be used to evaluate the chance of dozing in the daytime [36].

#### 2.8.3. Quality of Life

SF-36 is constructed to survey health status in the medical outcomes study [37]. It includes 36 self-report items regarding daytime functioning [38]. The items are grouped into 8 dimensions: physical functioning (PF), social functioning (SF), role physical (RP), bodily pain (BP), mental health (MH), role emotional (RE), vitality (VT), and general health (GH) [37].

The questionnaires of ESS, PSQI, and SF-36 used in the trial were Chinese versions proved to be reliable and valid in China [35, 39, 40].

### 2.9. Statistical Analysis

All analyses were performed on the intention-to-treat (ITT) population of participants who had at least one treatment. Missing data were replaced according to the principle of the last observation carried forward. The significance level used for statistical analysis with 2-tailed testing was 5%. Data values were presented by mean ± SD, 95% confidence intervals (CI), or percentage. 

We conducted chi-square test for the case of proportions and analyses of variance (ANOVA) for testing the baseline differences between treatment groups. For PSQI, ESS, and SF-36 scores, Mauchly's test of sphericity was applied to judge whether there were relations among the repeatedly measured data. If any *P* ≤ 0.05, multivariate analysis of variance (MANOVA) was performed and data in different groups of each measurement time were compared pairwise. The method of Bonferroni was used to do pairwise comparisons of the repeatedly measured data in different measurement times of each treated group. All analyses were performed using the Statistical Package for the Social Sciences (SPSS) version 11.5 statistics software.

## 3. Results

### 3.1. Study Population (Figure 1)

**Figure 1:**
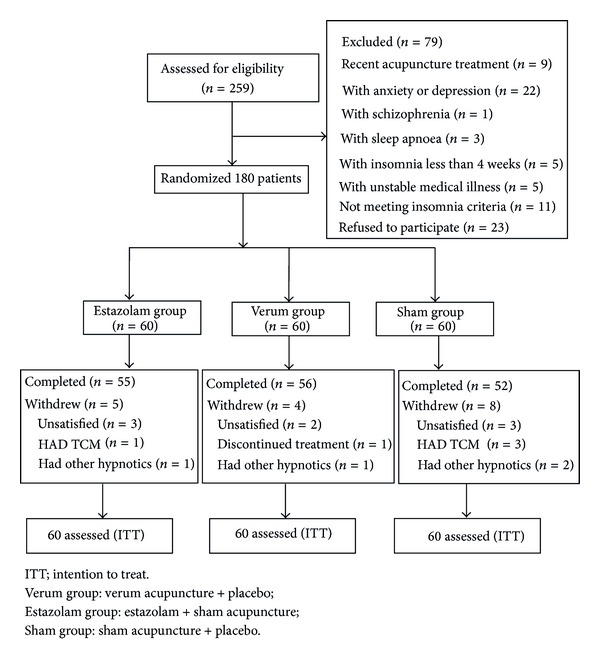
Trial profile.

A total of 259 participants were assessed for eligibility; 79 were excluded (23 refused to participate when informed the possibility of being assigned to the sham group, 56 were excluded for having depression, anxiety, schizophrenia, sleep apnoea, and other diseases). 180 patients were randomized to the verum group, estazolam group, or sham group (Figure 1).

Seventeen subjects (9.4%) withdrew during the study period, 5 (8.3%) from the verum acupuncture group, 4 (6.7%) from the estazolam group, and 8 (13.3%) from the sham group. None of the subjects withdrew due to adverse events.

*Demographic and Clinical Features (Table 1).* Table 1 presents the baseline characteristics. There were no significant differences identified in demographic and clinical features of the three groups. (The baseline data of SF-36 were listed in Table 2.)

### 3.2. Outcome Measurements 

#### 3.2.1. Sleep Measure: PSQI (Figure 2)

**Figure 2:**
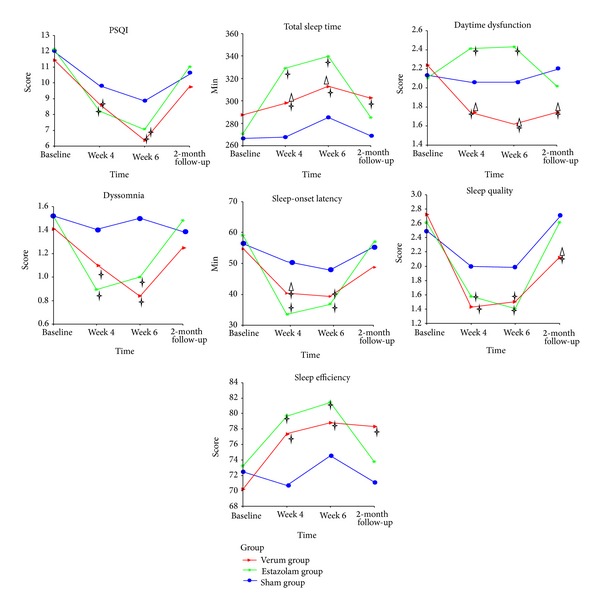
Change in Pittsburgh Sleep Quality Index and subscale scores at different times. Using repeated measures and multivariate analysis of variance (MANOVA) process of the general linear model and giving comparison among different groups and different measure time pairwise. ^†^ *P* < 0.05, versus sham acupuncture group at the same time point. ^△^ *P* < 0.05, versus estazolam group at the same time point.

Figure 2 presents changes of PSQI and subscales among the three groups. Compared with baseline, the verum, estazolam, and sham groups had better global score of PSQI and sleep quality, decreased sleep-onset latency and dyssomnia, longer sleep duration (only in the estazolam group), and higher sleep efficiency (not obvious in the sham group) (*P* < 0.05). Daytime dysfunction score increased in the estazolam group while decreased in the verum group (*P* < 0.05). However, most of the variables returned to baseline level at follow-up in the sham and estazolam groups. The significant differences in SQ, TST, SE and DD were well maintained to follow-up period in the verum group (*P* < 0.05). Subjects in verum group had lower DD and higher SQ scores than those receiving estazolam and sham acupuncture (*P* < 0.05). Both the verum and estazolam groups had significantly reduction in most of PSQI subscale scores at posttreatment compared with the sham group (*P* < 0.05). Whereas the difference in PSQI total score, SOL was not significant among the three groups at the 2-month follow-up.

#### 3.2.2. Daytime Functioning

**Figure 3:**
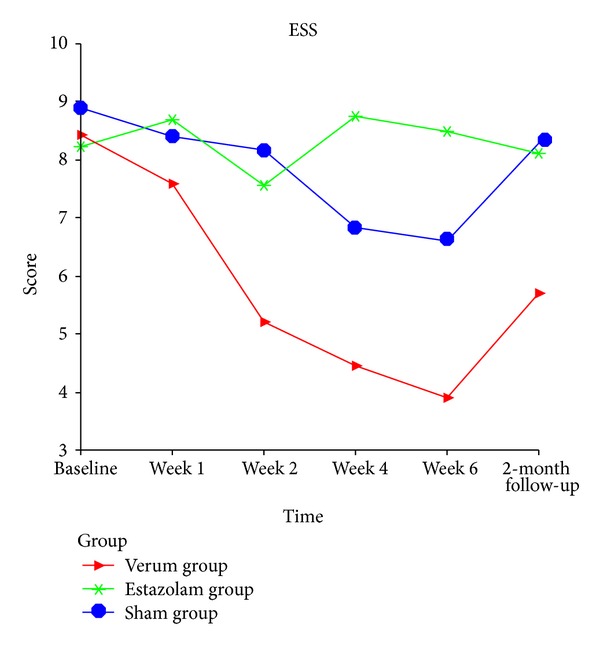
Change in Epworth Sleepiness Scale score from baseline to 2-month follow-up. Data from repeated measures and multivariate analysis of variance (MANOVA) process of the general linear model.

*Epworth Sleepiness Scale (ESS) (Figure 3)*. Figure 3 shows ESS data. There was significant decrease compared with baseline in the verum and sham groups. In the estazolam group, ESS score increased at the treatment phase and returned to baseline at follow-up. Compared with estazolam and sham groups, verum acupuncture group showed significant reduction in ESS score at the treatment and follow-up period (*P* < 0.05).

#### 3.2.3. Quality of Life

*SF-36 (Table 2).* Table 2 presents the data of SF-36. Compared with baseline, role physical and social functioning were improved in the verum and estazolam groups; role emotional was improved in the three groups (*P* < 0.05). The verum group reported greater feeling of vitality compared with baseline.

Verum acupuncture showed significant improvement in VT compared with the other two groups. Both the verum acupuncture and estazolam groups resulted in obvious improvement in SF, RE scores compared with the sham group (*P* < 0.05). 

#### 3.2.4. Other Clinical Outcomes

**Figure 4:**
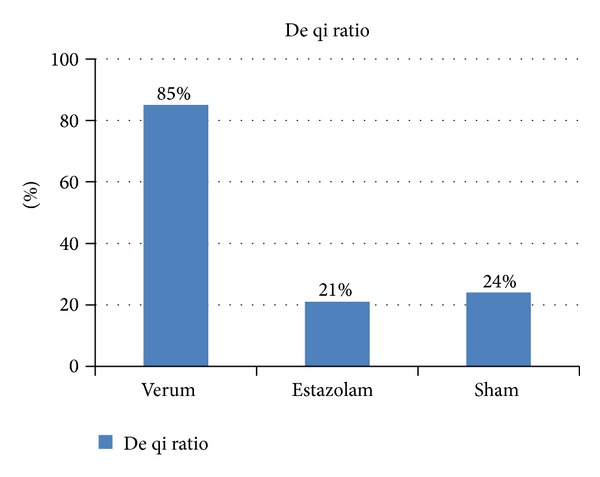
De qi ratio. De qi ratio means the ratio of De qi points to total points in three groups.

*(1) Needle Sensations Measuring (Figure 4)*. Figure 4 presents the ratio of De qi points to total points. The subjects were required to describe the needle sensation of every acupoint when needle manipulation was performed (tingling, burning, heaviness, fullness, numbness, soreness, and aching, etc.). De qi sensation was recorded “Y” and “N” for no obvious sensation. In the verum group, De qi sensation was obvious in 85% acupoints, while in the sham and estazolam groups De qi sensation was reported in only 24% and 21% acupoints. The results showed that De qi manipulation was well controlled in the trial.

*(2) Adverse Events (Table 3)*. In the verum acupuncture group 15 subjects developed local hematoma, 5 subjects complained of headache, and 5 subjects reported dizziness. In the estazolam group 18 subjects had local hematoma, 10 subjects reported headache, and 7 subjects developed dizziness. In the sham group 11 subjects developed local hematoma, 12 subjects reported headache, and 6 subjects developed dizziness. A total of 10 subjects had local muscle convulsion. All adverse events were mild. 

## 4. Discussion

This study was a double-dummy, single-blinded, randomized, placebo-controlled clinical trial. The aim was to investigate the efficacy of acupuncture in patients with primary insomnia.

The results of the present trial showed that all treatments were effective compared with pretreatment baseline. Improvements of sleep quality, total sleep time, sleep efficiency, daytime functioning achieved in the verum group were well maintained to follow-up, whereas the effect of sham acupuncture and estazolam was not significant when the intervention ended. 

Verum acupuncture was better than sham acupuncture and estazolam in improving sleep quality (at 2-month follow up). One of the most notable results of the trial was that verum acupuncture could significantly improve daytime functioning. Subjects in verum group achieved lower DD and ESS scores compared with the other two groups. They reached higher VT (the feeling full of energy [37]) scores at the same time. No significant within-group and between-group differences in PF, BP, and GH were detected. The possible reason might be that most of the subjects in our clinic were young and middle-aged and their health-related quality of life was not affected by insomnia critically. Thus, the SF-36 was not sensitive enough to detect the health status for insomnia on the cohort of patients recruited in our study.

Few studies used placebo acupuncture as comparison for investigating the efficacy of acupuncture in insomnia. The results of our trial can be compared with those obtained in an RCT by Yeung et al. [41], which compared electroacupuncture with placebo acupuncture. Compared with noninvasive placebo acupuncture, electroacupuncture showed statistically significant improvements in SE in their study. By contrast with the trial by Yeung et al., our data suggested that verum acupuncture could produce significant improvements in sleep quality, total sleep time, sleep efficiency, and daytime functioning than sham acupuncture. The differences might be due to different treatment durations (3 weeks versus 6 weeks), differences of the acupuncturists, acupoints, and needle manipulation procedures.

Double-dummy technique in our trial might also attributed to the difference. The technique was common in clinical drug trials [42, 43], and it has been tried in some clinical trials with acupuncture, for example, the trial which demonstrated the efficacy of acupuncture for migraine prophylaxis [44]. The design helped to increase compliance. In Chinese acupuncture clinic, it is difficult to only prescribe west medicine to a subject in a trial, which will result in high dropout rate. Double-dummy control of placebo medicine and sham acupuncture was applied in our trial to make blinding practicable. 

In our trial the function of De qi was considered. “De qi” was based on subjective reporting of the patient (soreness, numbness, fullness, radiating sensation, etc.) and was regarded as a sign of efficacy according to TCM. Most contemporary acupuncturists still seek De qi and believe it fundamental for efficacy [33]. Manual stimulation was applied to the verum group, and the results showed that De qi sensation was obvious in 85% acupoints, which ensured the efficacy of verum acupuncture. With nonspecific points and no manual stimulation, De qi sensation was reported only in 21–24% acupoints in the sham and estazolam groups. The placebo effect produced by sham acupuncture was considered to have less influence upon the disease, although needle pricking might induce nonspecific physiological reactions. The significantly improvements of verum acupuncture than sham acupuncture demonstrated the importance of De qi. 

According to TCM theory, the states of “energetic daytime function” and “powerful nocturnal sleep” form a circulation. If the circle is broken, the vicious spiral of “daytime low-spirit” and “nighttime hyperarousal state” will occur. Acupuncture is considered to play an important role in reestablishing the normal sleep-wake cycle. The result of the present study was in accordance with the theory. 

Points selection is crucial for efficiency. Based on literature review and TCM clinical experiences, Shenting (DU-24), Sishencong (EX-HN1), Baihui (DU-20), and Shenmen (HT-7) are most common in the treatment of insomnia, depression, anxiety, and so forth. Sanyinjiao (SP-6) is important to induce sedation and tranquilization. The points of sham group are mainly for local disease, having no relationship with treatment for insomnia. The results showed the overall effect of verum acupuncture on both nocturnal sleep and daytime functioning. 

As a benzodiazepine derivative, estazolam is efficacious in increasing sleeping time as well as reducing awakenings during the night [45]. It was chosen as the control drug for its wide applications in the treatment of insomnia in China. As hypnotic drugs are recommended to be used preferably intermittently rather than regularly [46], estazolam was given every other day in our trial.

The present study was limited by the lack of objective sleep assessments. It should be complement with multiple sleep latency test (MSLT), polysomnography or actigraph. One potential limitation was the lack of assessment of cognitive abilities problems (e.g., attention, concentration, and memory), for the related questionnaires (e.g., Dysfunctional Beliefs and Attitudes About Sleep Scale, the Pre-Sleep Arousal Scale, and the Sleep Hygiene Awareness and Practices Scale) were not widely used in China. Subgroups classification should also be considered, such as difficulty initiating and maintaining sleep or nonrestorative sleep. The previous study has suggested that some subgroups of patients with insomnia might be more inclined to increase sleepiness [38].

The present trial showed that ESS scores of estazolam group were not stable at the treatment time. It was considered that daytime somnolence was the most common adverse event of estazolam and would result in increased ESS scores. The somnolence effect usually ended by noon [47]. So different time points of assessment would lead to ESS score difference. The assessment time point should be taken into account in future research. 

In summary, our study presented some important data on the treatment of primary insomnia with acupuncture. The trial implied that verum acupuncture was superior in improving sleep quality and daytime functioning of primary insomnia compared with estazolam and sham acupuncture. Further research could be conducted with objective measure (PSG, MLST), subgroup design, and assessment of cognitive abilities problems. 

## Figures and Tables

**Table 1:** Demographic and clinical characteristics of the ITT population (Mean ± SD).

Variables	Verum group (*n* = 60)	Estazolam Group (*n* = 60)	Sham Group (*n* = 60)	*x* ^2^ *F* value	*P* value
Age, years	47.5 ± 13.3	50.1 ± 15.6	49.2 ± 12.0	0.54	0.59
Sex, male/female	19/41	21/39	18/42	0.36	0.84
Education attainment, y	10.2 ± 3.5	9.7 ± 4.2	11.4 ± 3.7	0.42	0.25
Insomnia duration, y	6.3 ± 2.1	5.7 ± 3.9	6.2 ± 4.8	0.33	0.57
Married	40	39	42	0.45	0.98
Widowed	9	10	9		
Single/separated/divorced	11	11	9		
Chronic medical illness	7	4	9	2.14	0.34
ESS	8.4 ± 2.7	8.2 ± 2.1	8.9 ± 2.2	1.26	0.29
PSQI total score	11.5 ± 2.0	12.1 ± 1.8	11.9 ± 2.2	1.42	0.24
SQ	2.8 ± 0.7	2.6 ± 0.6	2.5 ± 0.8	2.30	0.10
SOL (min)	55.5 ± 10.9	59.0 ± 13.1	56.0 ± 13.1	1.39	0.25
TST (min)	285.0 ± 54.5	273.0 ± 62.7	263.0 ± 59.1	2.10	0.12
SE (%)	70.2 ± 10.8	73.5 ± 8.9	71.6 ± 10.6	1.57	0.21
Dyssomnia	1.4 ± 0.7	1.5 ± 0.9	1.5 ± 0.8	0.33	0.72
DD	2.2 ± 0.7	2.1 ± 0.7	2.1 ± 0.6	0.53	0.59

ITT: intention to treat. verum group: verum acupuncture + placebo; estazolam group: estazolam + sham acupuncture; sham group: sham acupuncture + placebo.

ESS: Epworth Sleepiness Scale; PSQI: Pittsburgh Sleep Quality Index; SQ: sleep quality; SOL: sleep-onset latency; TST: total sleep time; SE: sleep efficiency; DD: daytime dysfunction; results from *x* ^2^ or ANOVA test for categorical and quantitative variables, respectively.

**Table 2:** Change in SF-36 (Mean ± SD) from baseline to 2-month follow-up.

Item timepoint	Verum group	Estazolam group	Sham group	*P*		
				Verum versus sham	Verum versus estazolam	Estazolam versus sham
PF						
Baseline	85.6 ± 10.8	88.1 ± 8.3	86.4 ± 10.4			
Week 6	88.4 ± 9.6	89.8 ± 6.4	87.7 ± 8.5	0.68	0.35	0.18
2-month follow-up	89.8 ± 7.7	87.9 ± 8.3	86.6 ± 9.1	0.05	0.24	0.42
RP						
Baseline	53.5 ± 10.0	55.1 ± 13.5	56.3 ± 10.9			
Week 6	63.1 ± 12.5*	64.2 ± 13.4*	59.7 ± 13.3	0.18	0.63	0.07
2-month follow-up	61.4 ± 14.3*	58.5 ± 16.7	57.2 ± 13.4	0.15	0.31	0.66
BP						
Baseline	84.7 ± 12.9	87.1 ± 8.0	85.3 ± 10.0			
Week 6	87.0 ± 13.6	89.7 ± 10.1	86.6 ± 11.3	0.85	0.24	0.18
2-month follow-up	86.3 ± 15.8	87.7 ± 13.8	83.5 ± 13.2	0.30	0.63	0.13
GH						
Baseline	35.7 ± 10.1	36.1 ± 8.6	33.2 ± 6.9			
Week 6	38.0 ± 10.2	36.0 ± 10.9	34.7 ± 10.6	0.11	0.31	0.53
2-month follow-up	36.6 ± 8.6	34.5 ± 10.7	33.8 ± 8.1	0.11	0.21	0.69
VT						
Baseline	36.8 ± 9.4	33.6 ± 9.9	34.4 ± 7.1			
Week 6	44.6 ± 13.1*	32.1 ± 10.1	37.7 ± 10.5	0.002	<0.001	0.01
2-month follow-up	42.3 ± 12.0*	32.1 ± 8.5	35.4 ± 10.0	0.001	<0.001	0.10
SF						
Baseline	73.6 ± 12.0	75.6 ± 9.7	72.1 ± 10.4			
Week 6	81.6 ± 11.5*	80.7 ± 17.0*	74.4 ± 15.7	0.02	0.75	0.03
2-month follow-up	76.3 ± 13.9	77.9 ± 15.6	73.0 ± 13.0	0.24	0.56	0.08
RE						
Baseline	31.3 ± 7.7	33.4 ± 6.3	32.3 ± 9.0			
Week 6	37.4 ± 19.5*	42.1 ± 16.5*	33.8 ± 17.0*	0.29	0.16	0.02
2-month follow-up	38.0 ± 16.4*	34.3 ± 14.3	31.8 ± 16.2	0.04	0.21	0.42
MH						
Baseline	48.1 ± 19.0	41.1 ± 18.4	47.5 ± 13.2			
Week 6	52.2 ± 17.9	54.3 ± 20.8*	48.6 ± 17.2	0.32	0.56	0.12
2-month follow-up	50.0 ± 19.3	48.6 ± 19.9	44.7 ± 14.6	0.14	0.70	0.27

SF-36: 36-item short-form health survey; MH: mental health; PF: physical functioning; RP: role-physical; BP: bodily pain; VT: vitality; GH: general health; SF: social functioning; RE: role-emotional.

Data from multivariate analysis of variance (MANOVA) and repeated measures. (Mauchly's test of sphericity: *P* < 0.05.) *Comparison within each group with baseline *P* < 0.05.

**Table 3:** Adverse events.

Symptom Group	Number	Severity	Disposal	Result
Local hematoma				
Verum	15	Mild	Cold compress	Reablement
Estazolam	18			
Sham	11			
Headache				
Verum	5	Mild	Resting	Reablement
Estazolam	10			
Sham	12			
Dizziness				
Verum	5	Mild	Resting	Reablement
Estazolam	7			
Sham	6			
Muscle convulsion				
Verum	2	Mild	Massage	Reablement
Estazolam	6			
Sham	2			
